# Cases in Surgery

**Published:** 1854-01

**Authors:** J. C. Hughes

**Affiliations:** Professor of Surgery in the Medical Department of the Iowa University


### *Cases in Surgery.*

BY J. C. HUGHES, M. D.,

Professor of Surgery in the Medical Department of the Iowa University.

Case I.—*Operation for Complicated Hare Lip.*—Thos. M'Guire, aged 18 years, presented himself, Nov. 29th, at the Hospital for operation. I found upon examination, his condition to be as follows: The external fissure, reaching continuously from the lower edge of the lip on the right side of the corresponding nostril, which was widely separated, being about an inch and a quarter wide at its lower or labial portion, and terminating in the nostril by an opening three quarters of an inch in width. There was also arrest of development in the superior maxillary, the fissure in the bones being partially filled by irregular teeth projecting horizontally from the left side. The complication with cleft palate which existed, extended through both the hard and soft structures, exceeding in width half an inch.

Preparatory to the operation for this repulsive deformity, I found it necessary to remove a number of the teeth which would obstruct the anticipated union of the soft parts. This having been done, forcible pressure was applied for some days to the projecting structures, for the purpose of obtaining a smooth basis for the parts about to be supported by them. These points having been accomplished, the patient was brought before the class for operation. After administering the the chloroform, (given at his own request,) I took hold of one side of the lip, separating it freely with the scalpel from its attachment with the gum, then adopting the same course with the other, both were made free; which is necessary to success, by diminishing the subsequent strain on the line of union. Then, to make a fresh surface upon the edges, I introduced a tenotomy knife through the lip at the nasal angle upon the left side of the fissure, making an incision obliquely outwards nearly as far as the labial border, when the direction was changed toward the fissured part. The same course was adopted with the opposite side, so that when the lips were brought together the projection formed by the angular incision would supply the deficiency in the lip, which would otherwise ensue. The angles of the lips were now brought upon the same level and accurately adjusted by passing a ligature through their lower edges. I then introduced a needle, bringing the raw edges in direct contact by the application of the twisted suture. The ligature was now removed and a second needle introduced, which was also retained by the twisted suture; after this the muscles were controlled by the isinglass plaster, which completed the operation.

The parts were kept at perfect rest for forty-eight hours; adhesion by the first intention took place, and on the third day the first needle was removed, without removing the suture; on the fourth day the second. The sutures and isinglass plasters were allowed to remain some eight days. Perfect and favorable union was the result.

The fissure of the cleft palate is so very wide any attempt to remedy it by a surgical operation is unfortunately out of the question. A metalic plate which is being fitted to the part by a dentist of this city, will obviate the difficulty as effectually as art can do it.

It is remarkable what a favorable effect the operation has had upon the appearance of the patient. The deformity had existed during his life time, and was accompanied by so marked a distortion of his features as to present an appearance almost revolting. At this time the nostril, formerly widely separated, is of a natural shape and width; the upper lip is of a uniform width and appearance, and the countenance of the patient shows no remains of his former misfortune. This contrast is more fully shown by the daguerreotypes in my possession, which give his appearance both before and after the operation. His enunciation also, formarly so indistinct and imperfect that strangers could not understand his half formed language, is now quite clear and distinct and can be readily understood.

Case II.—*Operation for false Anchylosis of the Inferior Maxillary, of over four years standing.*—Gertrude Van Ballegooyer, aged eleven years, entered the Hospital Saturday, Dec. 10th. More than four years ago, in consequence of injudicious mercurial treatment, severe and extensive ulceration of the right cheek was produced, resulting in the formation of extensive bands of adhesion between the whole of the inner structure of the cheek and the superior and inferior maxillaries. A portion of the muscular structure of the part having been destroyed and its place occupied by the new deposit contracted during the healing process, bringing the jaws in such close contact that a thin knife blade could not be passed between the teeth. Owing to this obstruction, the patient could take no solid food, and of course her only diet during the four years, consisted of various liquids which were taken into the mouth through an opening on its side caused by the absence of one or two teeth.

The adhesions began at the angles of the lips, extending backwards from the canine or first bicuspid tooth, a distance which could not be ascertained, as it was impossible to make an internal examination owing to the close proximity of the jaws and the adhesions, and an external touch gave only an indistinct means of diagnosis; yet it was the only source of information.

After all the examination by myself as well as my colleagues, which the case admitted of—the presumption being that the adhesion consisted of bands exclusively ligamentous—it was decided that the evil might be remedied by an operation.

The patient was now brought before the class for operation, and by the request of her friends placed under the partial influence of the anæsthetic agent.

I commenced the operation by inserting a straight and narrow bistoury into the angle of the mouth, passing it backwards to the distance of some two inches between the morbid adhesions and the sound structures of the cheek; then changing the position of its edge, cut inwards towards the line of separation between the jaws. But here the progress of the bistoury was unexpectedly arrested by what proved to be osseous deposit, a condition not anticipated. This caused the union to be doubly firm, and the operation much more difficult.

Resort was at once had to a straight and narrow saw, (more delicate than Barton's metacarpal saw,) which I introduced into the opening made by the bistoury, and succeeded in dividing the bony formations. The bistoury was again introduced, when the separation of the bands was partially completed, so that the jaws could be slightly separated. I then introduced between the teeth an instrument which I had had constructed for the purpose, the levers of which were made to diverge by the action of a screw upon the outside, producing a separation of the teeth to the distance of half an inch or more. After this separation, a further examination internally was practicable, and I found the ligamentous adhesions to extend as far back as the angle of the jaw. Farther incisions sufficient to separate the remaining adhesions, were made by a scalpel whose point was protected, and the edge of which was unusually convex. Fearing that contraction might again take place during the healing process, I made incisions in an oblique direction above and below the first, to the entire depth of the bands. The jaws could now be distended without difficulty, to the extent of one and a half or two inches, by the action of the screw.

Not much hemorrhage occurred during the operation, and it being now completed, the lever was removed from the mouth, slight distension being maintained by the insertion of a piece of cork between the teeth, when the parts were allowed to remain quiet for several hours. The lever was again introduced and the mouth fully distended. It was again removed, and the cork inserted during the night. The inflammatory excitement was controlled by the application of cloths wet with cold water. (The same course of treatment is still being continued.) The case at this writing is doing well, she having the power, without the aid of the lever, to distend her mouth to the extent of more than an inch.

I am not aware that any other case has been successfully operated upon in the west and recorded, where osseous union has existed.

